# Comparing accelerometer, pedometer and a questionnaire for measuring physical activity in bronchiectasis: a validity and feasibility study?

**DOI:** 10.1186/s12931-016-0497-2

**Published:** 2017-01-14

**Authors:** B. O’Neill, S. M. McDonough, J. J. Wilson, I. Bradbury, K. Hayes, A. Kirk, L. Kent, D. Cosgrove, J. M. Bradley, M. A. Tully

**Affiliations:** 1 Centre for Health and Rehabilitation Technologies, Institute for Nursing and Health Research, Ulster University, Newtownabbey, UK; 2 UKCRC Centre of Excellence for Public Health (Northern Ireland), Belfast, UK; 3 Centre for Public Health, School of Medicine, Dentistry and Biomedical Sciences, Queen’s University Belfast, Belfast, UK; 4 School of Psychological Sciences and Health, University of Strathclyde, Glasgow, UK; 5 Northern Ireland Clinical Research Network, Respiratory Health, Belfast Health and Social Care Trust, Belfast, UK; 6 Centre for Experimental Medicine, School of Medicine, Dentistry & Biomedical Sciences, Queen’s University Belfast, Belfast, UK

**Keywords:** Physical activity measurement, Bronchiectasis, Step count, Actigraph, Pedometer

## Abstract

**Background:**

There are challenges for researchers and clinicians to select the most appropriate physical activity tool, and a balance between precision and feasibility is needed. Currently it is unclear which physical activity tool should be used to assess physical activity in Bronchiectasis. The aim of this research is to compare assessment methods (pedometer and IPAQ) to our criterion method (ActiGraph) for the measurement of physical activity dimensions in Bronchiectasis (BE), and to assess their feasibility and acceptability.

**Methods:**

Patients in this analysis were enrolled in a cross-sectional study. The ActiGraph and pedometer were worn for seven consecutive days and the IPAQ was completed for the same period. Statistical analyses were performed using SPSS 20 (IBM). Descriptive statistics were used; the percentage agreement between ActiGraph and the other measures were calculated using limits of agreement. Feedback about the feasibility of the activity monitors and the IPAQ was obtained.

**Results:**

There were 55 (22 male) data sets available. For step count there was no significant difference between the ActiGraph and Pedometer, however, total physical activity time (mins) as recorded by the ActiGraph was significantly higher than the pedometer (mean ± SD, 232 (75) vs. 63 (32)). Levels of agreement between the two devices was very good for step count (97% agreement); and variation in the levels of agreement were within accepted limits of ±2 standard deviations from the mean value.

IPAQ reported more bouted- moderate - vigorous physical activity (MVPA) [mean, SD; 167(170) vs 6(9) mins/day], and significantly less sedentary time than ActiGraph [mean, SD; 362(115) vs 634(76) vmins/day]. There were low levels of agreement between the two tools (57% sedentary behaviour; 0% MVPA_10+_), with IPAQ under-reporting sedentary behaviour and over-reporting MVPA_10+_ compared to ActiGraph. The monitors were found to be feasible and acceptable by participants and researchers; while the IPAQ was accepta ble to use, most patients required assistance to complete it.

**Conclusions:**

Accurate measurement of physical activity is feasible in BE and will be valuable for future trials of therapeutic interventions. ActiGraph or pedometer could be used to measure simple daily step counts, but ActiGraph was superior as it measured intensity of physical activity and was a more precise measure of time spent walking. The IPAQ does not appear to represent an accurate measure of physical activity in this population.

**Trial registration:**

Clinical Trials Registration Number NCT01569009: Physical Activity in Bronchiectasis.

## Background

Global recommendations on physical activity for health have been developed to support the promotion of physical activity [1]. Similarly, the promotion of physical activity for patients with respiratory conditions is gaining momentum, and the measurement of physical activity is becoming more common in clinical practice [2–6]. Currently, it is unclear which physical activity tool should be used to assess physical activity in Bronchiectasis (BE). Research in other respiratory populations (COPD and CF) supports the use of triaxial accelerometers such as the ActiGraph; however, currently there is no published research on the best tool for the measurement of physical activity in BE [4, 7–9]. Questionnaires e.g. the International Physical Activity Questionnaire are also used for assessment of physical activity in respiratory populations [10]. A recent review on the validity of a range of additional questionnaires to measure physical activity in COPD has reported the lack of conceptual framework underpinning their development [11]. The value of monitors, such as ActiGraph, over subjective questionnaires is the provision of precise objective information on physical activity dimensions (frequency, duration, intensity and type), that is not limited by recall and response bias [12]. It is not clear if all these dimensions are useful or necessary, of if the high levels of precision are needed to detect differences in physical activity between groups or changes over time [13]. Other simple objective tools are available e.g. pedometers but the validity, feasibility and acceptability of these have not been compared to the ActiGraph in this population.

There are challenges for researchers and clinicians to select the most appropriate physical activity tool, and a balance between precision and feasibility is needed. Feasibility can be measured by examining the extent to which an instrument provides extra information not already available to the health care provider, the practicality of use within clinical practice with respect to the degree of training required, method of administration, ease of administration, analysis and interpretation and time taken to administer and score as well as acceptability of the device to patients and clinicians [14]. There is currently limited information about the feasibility and precision of monitors in BE. This information would be particularly useful for clinicians and researchers when deciding which instrument to choose.

## Methods

### Study aims

The aim of this research is to compare assessment methods for the measurement of physical activity dimensions in people with BE. Specifically we will (i) compare assessment tools (pedometer and IPAQ) to our criterion method (ActiGraph) for the measurement of physical activity dimensions, and (ii) assess the feasibility and acceptability of objective assessment tools (ActiGraph, pedometer) and subjective assessment tools (IPAQ) to assess physical activity dimensions in BE from two perspectives: those of the participants’ and those of the researchers’ conducting the outcome measurements.

### Study design

Patients in this analysis were enrolled in a cross-sectional study using quantitative methodology. Additional methods and results from the primary study which relates to patterns correlates of sedentary behaviour and physical activity have been published previously [15]. Participants who agreed to take part in the study attended a clinical visit (Visit 1) where they provided baseline demographics; disease severity was estimated using the Bronchiectasis Severity Index [16]. During this visit, an ActiGraph GT3X+ accelerometer and DigiWalker CW700 pedometer were attached and participants were asked to wear the devices for 7 consecutive days following this visit. Eight days later, participants attended for a second clinical visit (Visit 2) where they returned the activity monitors (ActiGraph and Pedometer) and completed the IPAQ and questionnaires relating to the feasibility and acceptability of the monitors/IPAQ.

### Setting and participants

Patients (*n* = 63) with bronchiectasis were recruited from the Northern Ireland Regional Respiratory Centre (Belfast City Hospital) (*n* = 23), Craigavon Area Hospital (*n* = 15) and Altnagelvin Area Hospital (*n* = 25) in one UK region (N Ireland). Inclusion criteria were patients aged ≥18 years, diagnosis of bronchiectasis confirmed by High Resolution Computerised Tomography or CT, clinically stable (no exacerbation and no significant change in symptoms or medication in the last four weeks) and sputum bacteriology completed over the past three months. Exclusion criteria were clinically unstable patients (pulmonary exacerbation or any change in symptoms or medication in the last four weeks), current severe haemoptysis, pregnancy or any other concomitant condition that would prevent participation. The study was approved by the NI Research Ethics Committees (Ethics Approval REC Reference: 12/NI/0044) and research departments of all the participating hospitals. Written informed consent was obtained from all of study participants. The study was supported by the Northern Ireland Clinical Research Network (NICRN) Respiratory Health.

### Study activity monitors

The physical activity dimensions for each of the instruments used are summarised in Table 1. At Visit 1, participants were offered a reminder system to receive daily, alternate-day or once-weekly reminders to wear their monitors. All devices were worn during all waking hours for seven consecutive full days following Visit 1; and any periods of non-wear-time were to be recorded in a daily ‘log’ provided at visit 1. The ActiGraph device and a sealed pedometer were placed beside one another on a belt, worn on the dominant side of the body in line with the anterior axillary line of the hip. As devices were not waterproof they were removed during water sports, washing or showering.

**Table 1:** Descriptors, categorisation and comparable dimensions of physical activity (PA) per instrument

Physical Activity Dimension	Descriptor	Categorisation of PA by activity monitor
Total physical activity time (mins/day)	Total time spent per day in physical activity at different intensities.	• ActiGraph total physical activity in minutes per day was based on ≥100 counts per minute (cpm) [29, 33] • ^a^Pedometer - total time spent in walking • IPAQ n/a
Steps per day	Total steps taken per day	• ActiGraph total daily step counts • ^a^Pedometer total daily step counts. A valid day of pedometer data required steps to lie between 100 and 50,000 steps. • IPAQ – n/a
Sedentary behaviour time (mins/day)	Average daily time spent at rest or inactive e.g. sitting/lying	• ActiGraph sedentary behaviour <100 cpm; [29, 33] • Pedometer – n/a • ^b^IPAQ - Time spent sitting
Light physical activity time (mins/day)	Average daily time spent in physical activity at a low intensity such as standing and slow walking around the home	• ActiGraph light physical activity 100–1951 cpm [33] • Pedometer – n/a • IPAQ – n/a
Total MVPA time (mins)	Average daily time spent in physical activity at a higher intensity such as brisk walking	• ActiGraph moderate-vigorous physical activity ≥1952 cpm [29, 33] • Pedometer – n/a • IPAQ- n/a
MVPA in ≥10-min bouts (mins/day)10+	Average daily time spent in 10 min bouts of physical activity at a higher intensity	• ActiGraph – moderate-vigorous physical activity ≥1952 cpm accumulated in ≥10-min bouts • Pedometer – n/a • ^b^IPAQ – Scores are usually reported as MET-mins, and we converted this to mins/day

n/a parameter not available

^a^pedometer dimensions comparable to ActiGraph

^b^IPAQ dimensions comparable to ActiGraph

#### ActiGraph

The ActiGraph GT3X+ (ActiGraph, Pensacola, Florida) is a tri-axial accelerometer that measures body acceleration as counts per unit time; using defined cut points for accelerometer counts it is possible to measure activity at different intensities e.g. light, moderate, vigorous intensity, as well as measure number of steps. The ActiGraph has been validated for measurement of physical activity dimensions in different populations, and as an accurate measure of sedentary behaviour [17, 18].

#### Yamax DigiWalker pedometer

The DigiWalker CW-700 is a pedometer which has been used in recent studies to measure step counts in the general population [19, 20], and also in respiratory populations such as COPD [21–23]. This has been shown to be the most reliable, commercially obtainable pedometer available [24]. This device does not measure intensity of physical activity.

### Study questionnaires

#### International Physical Activity Questionnaire (IPAQ)

The long-form IPAQ is a validated 27-item, self-completed physical activity questionnaire which was developed to measure health related physical activity (i.e. activity in bouts of at least 10 min) in most daily situations. It was chosen as it allows participants to include leisure/exercise, walking, occupation, and transportation physical activity in their responses, and the scoring protocol allows greater flexibility in how physical activity is accumulated than other questionnaires [10, 25]. The IPAQ identifies the frequency and duration of moderate and vigorous physical activity, walking physical activity, and time spent sitting during the past week in (i) work related physical activity (ii) transportation physical activity (iii) housework/house maintenance/caring for the family time physical activity and (iv) recreation/sport/leisure time physical activity. The IPAQ has been utilised in patients with COPD [26].

#### Feasibility questionnaires

Feasibility questionnaires for the study were developed to facilitate feedback from both participants and researchers about the activity monitors and the IPAQ. These were informed by questionnaires that were used in previous research exploring feasibility of accelerometers [27]. A 10-point visual analogue scale (VAS) (0 ‘very difficult/uncomfortable’ through to 10 ‘very easy/comfortable’) was used to assess participants’ perspectives on the ease of fitting, and comfort of wearing the ActiGraph/pedometer. Participants were also asked to report any difficulties relating to wearing the monitors, or any strategies they adopted to make it easier to wear the monitors.

Researchers’ perspectives on how easy it was to teach participants to apply and wear the monitors was assessed on a 10-point VAS (0 ‘very difficult’ through to 10 ‘very easy’), and they also recorded how long it took to download data. Both participants and researchers’ perspectives on ease of completing/administering and usefulness of the IPAQ was also assessed using a 10-point VAS (0 ‘very difficult/not useful’ through to 10 ‘very easy/very useful’).

### Data handling and analysis

For the ActiGraph and pedometer, participant wear time logs were cross-checked to explore periods of non-wear time of the monitors; for example, if the participant had removed the monitor for bathing or swimming during waking hours. Step count was classified into the following categories: sedentary <5000 steps per day, low active 5000–7499 steps per day, somewhat active/active ≥7500 steps per day [28].

Table 1 indicates the physical activity dimension variables that were compared between the ActiGraph and the pedometer or the IPAQ. Due to the limited number of participants performing vigorous physical activity, moderate and vigorous categories were combined. Thus, ActiGraph moderate - vigorous physical activity (MVPA) reflected time spent in moderate/vigorous activity and this included walking time [29]. The IPAQ records walking and moderate/vigorous activity domains separately so to aid comparison these were combined into a ‘single’ moderate/vigorous activity domain for analysis.

#### ActiGraph

ActiGraph data was considered valid if there were more than 600 min of monitoring per day and at least 5 days, one being a Saturday or Sunday, giving a sample with valid data of *n* = 55 [30, 31]. Using ActiLife software version 6.8.0, wear-time validation was applied using parameters set by Choi (2011) which allowed for a 2 min interval of non-zero counts with an up/downstream 30 min of consecutive zero counts window [32]. Step counts were calculated using cut-off points based on manufacturer guidelines [33]. Categorisation of physical activity dimensions from the ActiGraph is summarised in Table 1.

#### Pedometer

After the 7 day monitoring period, data collected from the pedometer on daily step counts and time spent in walking was recorded. A valid day of pedometer data required steps to lie between 100 and 50,000 steps (Table 1). Datasets which aligned with ActiGraph data (*n* = 50), with respect to wear time, were included in the analyses.

#### IPAQ

Scores are reported as METs (*n* = 55), one MET-minute is defined as the MET-intensity multiplied by the minutes per week of activity. MET-intensity levels used to score the IPAQ questions were vigorous (8.0 METs), vigorous chores (5.5 METs), moderate (4.0 METs), outside/yard chores (4.0 METs) inside chores (3.0 METs), cycling (6.0 METs) and walking (3.3 METs). Time spent sitting, walking, and in moderate, and vigorous activity was calculated along with walking, moderate and vigorous MET/mins. Physical activity categorical scores (low, moderate or high) were calculated [International Physical Activity Questionnaire 2005].

### Statistical analysis

Descriptive statistics were used to summarise study populations demographic and clinical characteristics. All data was transferred to SPSS for statistical analysis and all statistical analyses were performed using SPSS 20 (IBM), unless otherwise stated. Summary data is reported as Mean (SD) and statistical significance was assumed at *p* < 0.05.

To investigate validity of the IPAQ questionnaire and pedometer, the percentage agreement between ActiGraph and these other measures was calculated using limits of agreement [34]. ActiGraph was considered the criterion measure and a value close to or equal to 100%, with narrow limits of agreement (LOA) indicated higher levels of agreement between the two measures under investigation. Patterns in the plots were also explored to identify any systematic bias between the measurements [35].

## Results

### Demographics and physical activity data

There were 55 (22 male) data sets available for analysis (Table 2). The mean[SD] age of the participants was 63[10]years and disease severity was categorised as Stage 1 Mild 27[49]% and Stage 2 Moderate/severe 28[51]% (Bradley et al 2015). There were *n* = 55/55 datasets available for ActiGraph and IPAQ, *n* = 50/55 for pedometer (five datasets had less than 5 valid days of pedometer readings).

**Table 2:** Demographic and clinical characteristics of patients with bronchiectasis (*n* = 55)

Age (years)	63 (10)
Gender (male/female)	22 [40]/33 [60]
BMI (kg/m^2^)	27 (4)
FEV_1_ (% predicted)	76 (21)
FVC (% predicted)	94 (19)
Disease Severity (%)^a^	
Mild	27 [49]
Moderate	18 [33]
Severe	10 [18]
Smoking History:	
Never (%)	46 [84]
Ex-smoker (%)	9 [16]
Number of oral antibiotic courses within last year	3 (2)
Number of IV antibiotic courses within last year	0–3 (range)

Results are Mean (SD) or Frequency [%]

*Abbreviations*: *BMI* body mass index, *FEV*_*1*_ forced expiratory volume in 1 s (% predicted), *FVC* forced vital capacity (% predicted)

^a^Disease severity based on Bronchiectasis Severity Index [16]

ActiGraph (*n* = 50) versus Pedometer (*n* = 50).

Steps per day and total physical activity time (mins) and were compared between the ActiGraph and the Yamax pedometer (Table 3). In terms of steps per day, there was no significant difference between the two devices; however total physical activity time (mins) as recorded by the ActiGraph was significantly higher than the pedometer (mean ± SD, 232 (75) vs. 63 (32)).

**Table 3:** Physical activity (ActiGraph, pedometer and IPAQ) for participants

Physical Activity Instrument	Mean(SD)/Median (IQR)
ActiGraph (*n* = 55)	
Steps per day	6,001(2780)^a^
Total physical activity time(mins/day)	232(75)^a^
Average times in different PA intensities^d^ (mins/day) unbouted (cpm)	
Sedentary behaviour time	634(77)^b^
Light physical activity time	207(63)
Total MVPA time	25(20)
Weekly bout-time	44(64)
MVPA_10+_ (mins/day)	6(9)^b^
Pedometer (*n* = 50)	
Daily step counts	6193 (3356)^a^
Total physical activity time (mins/day)	63 (32)^a^
IPAQ (*n* = 55)	
Total PA level (MET-min/week)	2700 (1037–6209)^c^
Walking (MET-min/week)	561 (165–1733)^c^
Moderate (MET-min/week)	1620 (450–4230)^c^
Vigorous (MET-min/week)	0 (0–480)^c^
Sedentary behaviour time (mins/day)	360 (270–437)^c^
	*362(115)* ^b^
MVPA_10+_ (mins/day)	99 (43–251)^c^ *167(170)* ^b^

*Abbreviations*: *cpm* counts per minute, *IPAQ* International Physical Activity Questionnaire, *MVPA* moderate-vigorous physical activity, *MVPA*_*10+*_ MVPA accumulated in ≥10-min bouts

^a^pedometer dimensions comparable to ActiGraph

^b^IPAQ dimensions comparable to ActiGraph

Results are Mean(SD) or Freq [%] or ^c^results given as median (IQR)

^d^ActiGraph PA category Sedentary <5000, Low active 5000–7499, Somewhat active and above >7500 steps per day

**Fig. 1:**
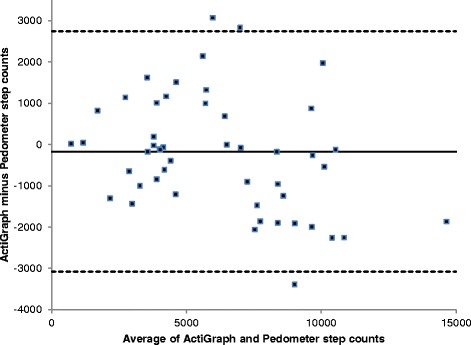
Bland-Altman Plot of ActiGraph vs Pedometer step counts. The mean (SD) difference in steps was -167 (1485), and the upper and lower LOA (2745, -3078)

**Fig. 2:**
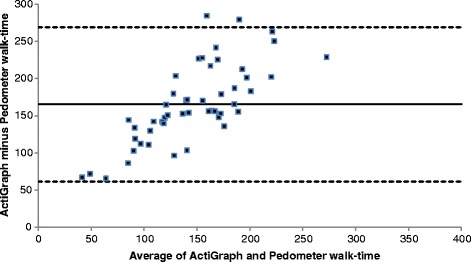
Bland-Altman Plot of ActiGraph vs Pedometer walk time. The mean (SD) difference in walk time was 165 (53) minutes and the upper and lower LOA (269, 62)

The Bland and Altman plots (Fig. 1) showed that the mean levels of agreement between the two devices was very good for step count (97% agreement); and although the individual differences between the devices were within acceptable limits for all but 3 participants (Fig. 1), the 95% LOA were wide and for some individuals this could vary by as much as 5823 steps. With respect to total physical activity time (mins) there was a lower level of agreement (28%, Fig. 2), with the pedometer under reporting total physical activity (or steps) time by 165 mins (or 2.75 h); and this was proportionally greater in people who were more active, so that the under reporting could be a great as 234 min (nearly 4 h).

ActiGraph (*n* = 55) versus IPAQ (*n* = 55).

IPAQ reported significantly less sedentary time than ActiGraph [mean, SD; 362(115) vs 634(76) vmins/day], and a much greater number of bouts of at least 10 min of -MVPA_10+_ [mean, SD; 167(170) vs 6(9) mins/day] (Table 3).

**Fig. 3:**
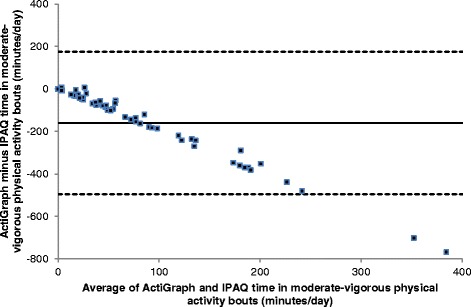
Bland-Altman Plot of ActiGraph vs IPAQ time in moderate-vigorous physical activity bouts (MVPA_10+_). The mean (SD) difference in bouts was 272 (135), and the upper and lower LOA (536, 7)

**Fig. 4:**
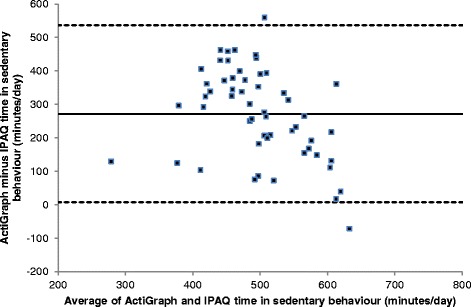
Bland-Altman Plot of ActiGraph vs IPAQ time in sedentary behaviour. The mean (SD) difference in sedentary behaviour was -160 (171) minutes, and upper and lower LOA (175, -495)

The Bland and Altman plots (Figs. 3 and 4) showed low levels of agreement between the two tools (57% sedentary behaviour; 0% MVPA_10+_), with IPAQ under-reporting sedentary behaviour and over-reporting MVPA_10+_ compared to ActiGraph. The latter finding showed bias, in that the over-reporting with IPAQ was greater in people who were more active; people who were very inactive were less likely to over-report MVPA_10+_.

### Categorising activity levels

It was possible to categorise patients into either ‘meeting’ or ‘not meeting’ the volume guidelines (≥150mins of at least moderate physical activity per week) or step guidelines (>10,000 steps per day) [28]. For the volume guidelines, 11% of patients met these guidelines according to ActiGraph compared to 82% with IPAQ. For the step guidelines, 7% (ActiGraph) compared to 14% (pedometer) respectively met these step-based recommendations.

A more detailed categorisation was possible for all 3 measurement tools. Using daily step count categories (see Data Handling and Analysis) and the ActiGraph data: 42% (*n* = 23/55) were categorised as sedentary, 29% (*n* = 16/55) low active and 29% (*n* = 16) somewhat active and above. Using daily step count categories and the pedometer step data (48%) *n* = 24/50 were categorised as sedentary, 14% (*n* = 7/50) low active and 38% (*n* = 19/50) somewhat active and above. The IPAQ scoring classification differs and IPAQ classified 18% (*n* = 10) of patients in low, 38% (*n* = 21) in moderate and 44% (*n* = 24) in high physical activity categories.

### Feasibility and acceptability

#### Participants

Feasibility and acceptability of monitors revealed that participants found them easy to fit: VAS (out of 10) mean ± SD score ActiGraph 9.8(0.76); pedometer 9.8 (0.76), and comfortable to wear VAS mean score mean score ActiGraph 9.22(1.03); pedometer 9.11 (1.24). Difficulties common to both ActiGraph (25%; *n* = 14) and pedometer (34%; *n* = 17) related to the elastic belt, on which both devices were located, becoming loose and slipping from its original location, the belt twisting on strenuous movement or difficulties opening and closing the belt clasp. One additional difficulty specific to the pedometer included discomfort and irritation from the metal clip against the body (2% *n* = 1/50). Strategies adopted by participants to make it easier to wear the ActiGraph and pedometer on the belt included wearing it next to their body as opposed to over clothing or vice versa to ensure a secure fit, and wearing the belt through trouser belt-loops to ensure a secure fit. To alleviate any skin irritation from the pedometer clip strategies included placing cotton between the pedometer clip and skin.

Feasibility and acceptability of IPAQ revealed that participants reported that it was easy to understand the questionnaire: VAS mean ± SD score 7.4 (2.03) and found it useful in its measurement of everyday activity levels: VAS mean score 7.64 (2.30). The average time to complete IPAQ was 12(6) minutes.

#### Researchers

Researchers reported that it was easy to teach participants how to apply and wear the monitors: VAS (out of 10) mean ± SD score ActiGraph 9.42(1.07); pedometer 9.29 (1.21). 13 participants requested one or more reminders as a prompt to wear the activity monitor belt. Downloading of the data by researchers took less time for the pedometer compared to the ActiGraph (1.5 min compared to just under 5 mins).

Researchers reported that while it was easy to administer the questionnaire, VAS mean score 7.87 (1.72), they noted that 48/55 (87.3%) participants required assistance to complete the questionnaire. Reasons for assistance included additional explanation or clarification of question terminology (24%); some participants required clarification of physical activity categories (69%) e.g. were unsure which physical activities should be included in different categories; some required clarification of the scoring of their answers (20%) or prompting to complete missing answers (44%); ‘other’ aspects (11%) included patients having difficulty recalling the week’s physical activity and they suggested future inclusion of a diary in the preceding week to act as an aide memoire.

## Discussion

This study aimed to compare two assessment tools (pedometer and IPAQ), with our criterion measure (the ActiGraph) to measure physical activity dimensions. In general, we showed that when measuring mean step count for a group of people either ActiGraph or pedometer could be used. However, when comparing individual step counts or when classifying physical activity according to step categories the devices were not interchangeable, as different step values were obtained from the ActiGraph and the pedometer within the same individual when worn for the same period of time. In addition, time spent in physical activity did not compare well between these two objective tools. The IPAQ does not appear to represent an accurate measure of physical activity in this population. All three tools appeared to be generally feasible and acceptable to patients and researchers.

The information from this study will help clinicians and researchers to make an informed decision about which tool to use. ActiGraph provides an advantage compared to the pedometer when a precise profile for patterns and intensity of physical activity dimensions is required e.g. total time spent in light physical activity, number of daily bouts of MVPA or sedentary periods [36]. However, the ActiGraph devices are significantly more expensive than pedometers (approximately $225 versus $20) and have a higher resource burden as qualified personnel are needed to manage and analyse this more complex data. If the primary outcome of interest is to change step counts in a group of people, as opposed to change intensity of physical activity, then a pedometer is a valid choice given the very high levels of agreement between group means for ActiGraph and pedometer step counts; this has been shown in other populations also [37]. Pedometers could be a cost effective option in large studies [38], as they are significantly less expensive than accelerometers; and resources such as specialised personnel or data management expertise are not required for the analysis of their simple step count outputs.

The low levels of agreement between the two devices for an individual is notable, and is in agreement with previous studies [38–40]. For example, in the study by Harris et al, 2009 in 121 older adults the LOA for an individual was approximately 8000 step while Kinnunen et al, 2011 reported a LOA of approximately 5000 steps [38, 40]. The reason for the wide variation in step counts between the two devices, demonstrated in our data, is not clear but suggests that the two assessment tools are not interchangeable; this is also suggested by Barriera et al. [37]. The devices measure movement in different ways; e.g. ActiGraph contains a triaxial electronic accelerometer and the pedometer is a uniaxial mechanical device, and such differences in technical specifications may impact on the validity of monitors [41, 42]. The lack of agreement in total physical activity time between the objective devices in our study remains unclear.

There is increasing interest in the measurement of sedentary behaviour resulting from evidence that it may be a distinct risk factor, independent of physical activity, for multiple adverse health outcomes in adults [43]. In our study, the ActiGraph and IPAQ assessment tools were not comparable in their measurement of either of these behaviours; the IPAQ underreported sedentary behaviour as well as over-reporting 10 min bouts of MVPA. These findings concur with a large population study of 1751 adults aged between 19–84 years which showed the same findings when comparing these two tools [44]; and can be explained by an increasing body of evidence that questionnaires do not relate accurately to objective physical activity assessment as their use is limited by recall and response bias and lack of precision/accuracy of the dimension data [4, 12, 45]. Conceptual frameworks for physical activity measurement propose a combined approach of direct and self-report (questionnaire) to enable a comprehensive exploration of physical activity patterns and behaviour [7, 46, 47].

The additional assessment of the feasibility of using different physical activity instruments in this study is important [7]. Both the ActiGraph and pedometer were feasible and acceptable to researchers in terms of their use and downloading data, with shorter data download time required for the pedometer as it provided less physical activity outputs; both monitors were acceptable to patients. While patients in this study indicated that the IPAQ was acceptable, most patients required assistance to complete it and therefore even though it is reportedly self-completed there needs to be a clinician/researcher present during completion to minimise misinterpreted or missing responses.

Research into the measurement of physical activity is expanding in other populations and suggestions for key factors to consider when selecting physical activity assessment tools include (a) the specific component of physical activity of interest; (b) the population; (c) cost and ease measurement; and (d) precision of measurement that is required [7]. A limitation of our study may be the positioning of the pedometer on the ActiGraph belt, which proved slightly problematic on some occasions and may have resulted in less step counts/walk-time being recorded in some people. Also we based our feasibility questionnaire around similar themes and response format to Hale et al. 2008, but such questions have not been validated for this specific use. However, to date research on the measurement on physical activity in bronchiectasis is limited, and this is the first study to assess multiple activity monitors in bronchiectasis. This study will help future research and clinical practice in bronchiectasis by providing data on the use and feasibility of physical activity measurement with ActiGraph, pedometer and IPAQ.

## Conclusion

Accurate measurement of physical activity is feasible in BE and will be valuable for future trials of therapeutic interventions. ActiGraph or pedometer could be used to measure simple daily step counts in BE, but ActiGraph was superior as it measured intensity of physical activity and was a more precise measure of time spent walking. The IPAQ does not appear to represent an accurate measure of physical activity in this population. The information reported in this study will be valuable for future trials of physical activity and sedentary behaviour patterns, and interventions. Future research could explore the application of a monitor like the ActiGraph, combined with a new validated and relevant self-report physical activity questionnaire in BE.

## Abbreviations

cpm: Counts per minute
IPAQ: International Physical Activity Questionnaire
IQR: Interquartile range
MVPA: Moderate-vigorous physical activity
MVPA_10+_: MVPA accumulated in ≥10-minute bouts
n/a: Not available
PA: Physical activity
SD: Standard deviation
